# Physical activity improves outcomes of combined lenvatinib plus anti-PD-1 therapy in unresectable hepatocellular carcinoma: a retrospective study and mouse model

**DOI:** 10.1186/s40164-022-00275-0

**Published:** 2022-04-04

**Authors:** Xue-Feng Liu, Xiao-Dong Zhu, Long-Hai Feng, Xiao-Long Li, Bin Xu, Kang-Shuai Li, Nan Xiao, Ming Lei, Hui-Chuan Sun, Zhao-You Tang

**Affiliations:** 1 Department of Liver Surgery and Transplantation, Liver Cancer Institute and Zhongshan Hospital, Fudan University, No. 180, Fenglin Road, Shanghai, 200032 China; 2 Key Laboratory of Carcinogenesis and Cancer Invasion of Ministry of Education, Shanghai, 200032 China; 3 Department of Hepatic Surgery, Fudan University Shanghai Cancer Center, Shanghai Medical College, Fudan University, Shanghai, 200032 China; 4 Department of Thoracic Surgery, Shanghai General Hospital, Shanghai Jiao Tong University, Shanghai, 200080 China; 5 Department of Hepatobiliary Surgery, Qilu Hospital of Shandong University, Jinan, 250012 China; 6 Department of Breast Surgery, Xin Hua Hospital Affiliated to Shanghai Jiao Tong University School of Medicine, Shanghai, 200092 China

**Keywords:** Hepatocellular carcinoma, Physical activity, Anti-PD-1, Lenvatinib

## Abstract

**Background:**

Physical activity is known to have anti-cancer effects, including immunomodulatory actions. This study investigated the hypothesis that physical activity synergizes with combined lenvatinib plus anti-PD-1 therapy to enhance efficacy in patients with unresectable HCC.

**Methods:**

The physical activity levels of patients with unresectable HCC receiving combined lenvatinib plus anti-PD-1 therapy were recorded by questionnaire. Patients were categorized according to physical activity levels (active vs. sedentary). The primary outcome was overall survival (OS). Secondary outcomes included objective response rate (ORR) and progression-free survival (PFS). A subcutaneous syngeneic HCC model was generated in C57BL/6 mice. Mice were randomized to receive placebo, combined lenvatinib plus anti-PD-1 antibodies or combination therapy plus physical activity. Tumors were measured every 3 days and harvested for immunohistochemistry analysis at 20 mm maximum diameter.

**Results:**

Fifty-nine patients with unresectable HCC were categorized to active (n = 28) or sedentary (n = 31) groups. The active group had higher albumin and des-γ-carboxy prothrombin levels and lower hepatitis B virus load at baseline; other clinical and oncologic characteristics were comparable between the two groups. Patients in the active group had significantly longer OS (HR = 0.220, 95% CI 0.060–0.799) and PFS (HR = 0.158, 95% CI 0.044–0.562) and higher ORR (OR = 4.571, 95% CI 1.482–14.102) than patients in the sedentary group. Regular physical activity was independently associated with OS, PFS and ORR. The mouse model showed that physical activity significantly suppressed tumor growth and prolonged survival of tumor-bearing mice. Furthermore, physical activity inhibited Treg cell infiltration and immune checkpoint expression (including CTLA4, TIGIT and TIM3) induced by long-term combined lenvatinib plus anti-PD-1 therapy, improving efficacy.

**Conclusions:**

Regular physical activity was associated with improved outcomes in unresectable HCC receiving combined lenvatinib plus anti-PD-1 therapy. Physical activity may improve therapeutic efficacy by reprograming the tumor microenvironment from an immunosuppressive to immunostimulatory phenotype.

**Supplementary Information:**

The online version contains supplementary material available at 10.1186/s40164-022-00275-0.

## Background

Primary liver cancer is the fifth most commonly diagnosed cancer and second leading cause of cancer-related death in China [1]. Hepatocellular carcinoma (HCC) represents 75–85% of primary liver cancer and is a major global health problem [2]. The majority of patients with HCC are diagnosed at an advanced stage, resulting in limited treatment options and poor prognosis [3]. Systemic therapy, including sorafenib, lenvatinib, and immune check point blockade (ICB), should be considered in patients with unresectable HCC. However, none of these treatment options have achieved satisfactory efficacy when used as monotherapy in published trials [4–8]. Recently, a phase 1b trial testing the combination of lenvatinib and pembrolizumab has shown encouraging results, with a response rate of 36% reported [9]. Nevertheless, adaptive resistance is still one of the major hinderances for those patients.

Upregulation of alternative immune checkpoints is considered one mechanism underlying adaptive immune resistance [10]. When resistance to anti-programmed cell death protein 1 (anti-PD-1)/anti-programmed cell death ligand 1 (anti-PD-L1) therapy develops, the proportion of T cells expressing alternative immune checkpoints, including cytotoxic T-lymphocyte-associated protein 4 (CTLA4) and T-cell immunoglobulin mucin-3 (TIM3), increases [11, 12]. Regulatory T (Treg) cells play a pivotal role in both maintaining immune homeostasis and tumor immune escape [13]. Moreover, PD-1 blockade significantly enhances the infiltration of immunosuppressive Treg cells, contributing to adaptive resistance to immunotherapy [14]. It is reported that lenvatinib reduces Treg cell infiltration and activates immune pathways, resulting in reprograming of the tumor microenvironment, which may contribute to improvement of the efficacy of anti-PD-1 therapy [15, 16]. Suppressing the infiltration of Treg cells and alternative immune checkpoints level is therefore an attractive strategy to improve the therapeutic efficacy of combined lenvatinib and anti-PD-1 antibody (Ab) therapy.

Regular physical activity is associated with a lower risk of developing several cancers, including HCC [17, 18]. In addition, physical activity reduces the risk of total deaths and cancer-related deaths in both colon and breast cancer [19–21]. Physical activity may reduce tumor cell proliferation, suppress epithelial-mesenchymal transition (EMT), and promote intra-tumoral perfusion/vascularization [22–24]. Moreover, physical activity elicits anticancer effects by reducing systemic inflammation and countering immunosenescence [25, 26]. We hypothesize that physical activity may synergize with combined lenvatinib plus anti-PD-1 therapy through immunomodulatory effects to enhance the therapeutic efficacy of this treatment regimen.

## Methods

### Patients

Patients with unresectable HCC treated with combined lenvatinib plus anti-PD-1 antibody at Zhongshan Hospital from June 1, 2018 to September 30, 2020 were included in this retrospective study. Inclusion criteria were as follows: (1) patients met the clinical diagnostic criteria for HCC [27], with or without pathological diagnosis; (2) HCC was unresectable and not suitable for transarterial chemoembolization (TACE); (3) treatment with lenvatinib combined with anti-PD-1 antibody was given as first-line systemic therapy; and, (4) an Eastern Cooperative Oncology Group (ECOG) performance status of 0 or 1. Patients were excluded if they: (1) had other malignancies; (2) received previous systemic treatment; (3) had no tumor evaluation after initiation of the combined treatment regimen; (4) had incomplete basic information; (5) were unable to specify physical activity details. This study was approved by the Hospital Research Ethics Committee. Informed consent was obtained from all patients.

### Grouping and measurement of physical activity

Patients took regular physical activity during combined lenvatinib plus anti-PD-1 treatment were classified into the active group, otherwise they were classified into the sedentary group. Physical activity was measured by questionnaire as previously described [19, 28, 29]. Patients or their immediate family members were questioned by phone survey in December, 2020. They were asked the following questions: (1) Does patient take any leisure time physical activity during pharmacotherapy? (2) What kind of physical activity does patient usually take? (brisk-walk, swimming, ball games, Tai Chi, or other aerobic activity) (3) How many days per week does patient take leisure time activity? (4) What is patient’s total time (minutes) of leisure time physical activity per day? (5) On a scale of 1 to 10, please rate the intensity of patient’s leisure time activity (perceived scale: 1–4 = low intensity, 5–6 = moderate, 7–10 = vigorous). The criteria for regular physical activity were based on the American College of Sports Medicine guidelines [30]. Patients were considered to engage in regular physical activity if they met any of the following criteria: (1) no less than 5 d·wk^−1^ of moderate aerobic activity for ≥ 30 min·d^−1^; (2) no less than 3 d·wk^−1^ of vigorous aerobic activity for ≥ 30 min·d^−1^; (3) no less than 3–5 d·wk^−1^ of mixed intensity activity for ≥ 30 min·d^−1^; or (4) any of the above before or within 1 month after the initiation of combination therapy until 2 months before death or the phone call.

### Treatment outcomes

The primary treatment outcome was overall survival (OS). Secondary outcomes included objective response rate (ORR) and progression-free survival (PFS). All outcomes were assessed using magnetic resonance imaging and modified response evaluation criteria in solid tumors (mRECIST) criteria for hepatocellular carcinoma response assessment [31].

### Cells and reagents

Hepa1-6 cells, derived from the BW7756 tumor in a C57L mouse, were obtained from the American Type Culture Collection (ATCC) (Manassas, VA, USA). The cells were cultured in high-glucose Dulbecco’s modified eagle medium (DMEM) (BasalMedia Technologies Co., Shanghai, China) supplemented with 10% fetal bovine serum (FBS) (Yeasen Biotechnology Co., Shanghai, China) and 1% penicillin–streptomycin (Yeasen Biotechnology Co., Shanghai, China) at 37 °C under a 5% CO_2_ atmosphere.

Lenvatinib was obtained from Eisai (Ibaraki, Japan). Anti-mouse PD-1 Ab (anti-PD-1 Ab; clone RMP1- 14) and mouse isotype control IgG (control IgG; clone 2A3) were purchased from Bio X Cell (West Lebanon, NH, USA).

Primary Abs used for immunohistochemical staining included: anti-CD4 Ab (#25229, CST), anti-CD8α Ab (#98941,CST), anti-Forkhead box protein p3 (Foxp3) Ab (ab253297, Abcam), anti-F4/80 Ab (#70076, CST), anti-CTLA4 Ab (A00020, Boster), anti-T cell immunoreceptor with Ig and immunoreceptor tyrosine-based inhibitory motif (ITIM) domains (TIGIT) Ab (A01962, Boster), anti-TIM3 Ab (ab241332, Abcam) and, anti-V domain-containing Ig suppressor of T-cell activation (VISTA) Ab (#54979, CST).

### Subcutaneous syngeneic mouse model

A subcutaneous syngeneic HCC model was generated by subcutaneously injecting ~ 3*10^6^ Hep1-6 cells in 100 μl PBS on the backs of 5–6-week-old female C57BL/6 J mice (Charles River Laboratories) (n = 21). Animals were weighed and tumor volume was assessed every three days. Once the maximum diameters reached 10 mm, animals were randomly assigned to receive combination therapy (lenvatinib plus anti-PD-1 Ab; n = 7), combination therapy plus physical activity (n = 7), or placebo (control, drug vehicle plus mouse isotype IgG; n = 7).

Lenvatinib (dissolved in 3 mM HCl, 10 mg/kg) was administered daily by oral gavage [32]. Anti-PD-1 Ab (200 μg/mouse) was intraperitoneally administered every five days [33]. Physical activity was facilitated by placing running wheels in cages; running distance was recorded by electromagnetic sensors in the combination therapy plus physical activity group. Mice were sacrificed and tumors harvested once tumor maximum diameter reached 20 mm. The study was performed in compliance with guidelines for the use of animals established by the institution ethical committee and the “Tumor induction in mice and rats IACUC Guideline” [34].

### Immunohistochemical staining and analysis

Immunohistochemical staining was conducted as previously described [35, 36] on tissue microarray. Images were captured using the PANNORAMIC panoramic slice scanner after staining. The Densito quant module in the Quant Center2.1 analysis software was used to quantify the H-Score (H-SCORE = ∑ (PI × I) = (percentage of cells of weak intensity × 1) + (percentage of cells of moderate intensity × 2) + (percentage of cells of strong intensity × 3), where PI represented the percentage of positive signal pixel area; I represented the coloring intensity).

Tumor viability, defined as the proportion of tumors presenting viable cells in a sample (i.e. excluding necrotic regions or granulation tissue), was assessed on hematoxylin and eosin slides. All analyses were performed by an expert pathologist blinded to the treatment arms.

### Statistical analysis

All statistical analyses were performed using SPSS (Version 22, Chicago, IL, USA). Continuous variables were compared with the Student’s t-test (equal variances assumed) or Wilcoxon rank sum test (equal variances not assumed). Categorical variables were analyzed by Chi-squared test or Fisher exact test. Kaplan–Meier survival analysis with the log-rank test was used to evaluate the associations between various interventions and survival. Univariate and multivariate logistic regression was used to determine factors affecting ORR. Multivariate Cox regression was used to determine variables associated with survival. For the analysis, a p-value < 0.05 in a two-tailed test was considered statistically significant. Graphs were drawn with Graphpad Prism (Version 8.0.2, San Diego, CA, USA).

## Results

### Baseline characteristics of patients

Fifty-nine patients were eligible for this study. Twenty-eight patients were classified into the active group and 31 into the sedentary group. Up to June 30, 2021, the median follow-up duration was 13 months (range, 3–28 months). The baseline clinical characteristics are summarized in Table 1. Compared to patients in the sedentary group, patients in the active group had higher albumin and des-γ-carboxy prothrombin (DCP) levels and lower hepatitis B virus (HBV) viral load at baseline (P < 0.05). However, the usage of antiviral drugs showed no significant difference between the two groups (0.796). Other clinical characteristics were comparable between the two groups.

**Table 1:** Baseline characteristics

Variables	Sedentary group (n = 31)	Active group (n = 28)	P value
Age (years), mean ± SD	55.9 ± 12.4	53.5 ± 8.1	0.387
Gender			
Male	26 (83.9%)	27 (96.4%)	0.245
Female	5 (16.1%)	1 (3.6%)	
PS score			
0	12 (38.7%)	18 (64.3%)	0.050
1	19 (61.3%)	10 (35.7%)	
Overweight			
No	30 (96.8%)	26 (92.9%)	0.599
Yes	1 (3.2%)	2 (7.1%)	
Child–Pugh classification			
A	30 (96.8%)	27 (96.4%)	1.000
B	1 (3.2%)	1 (3.6%)	
BCLC stage			
A	1 (3.2%)	3 (10.7%)	0.520
B	7 (22.6%)	6 (21.4%)	
C	23 (74.2%)	19 (67.9%)	
CNLC stage			
I	1 (3.2%)	3 (10.7%)	0.520
II	7 (22.6%)	6 (21.4%)	
III	23 (74.2%)	19 (67.9%)	
Tumor size (cm), mean ± SD	12.3 ± 5.5	10.7 ± 5.8	0.351
Extra-hepatic metastasis			
No	26 (83.9%)	21 (75.0%)	0.398
Yes	5 (16.1%)	7 (25.0%)	
Macrovascular invasion			
No	11 (35.5%)	14 (50.0%)	0.260
Yes	20 (64.5%)	14 (50.0%)	
WBC (× 10^9^/L), mean ± SD	5.8 ± 2.0	5.7 ± 1.9	0.828
NLR, mean ± SD	3.3 ± 1.6	2.7 ± 1.2	0.124
TB (μmol/L), mean ± SD	17.5 ± 9.6	16.7 ± 7.6	0.753
ALB (g/L), mean ± SD	38.5 ± 5.8	41.6 ± 4.0	0.020
ALT (U/L), mean ± SD	42.0 ± 26.3	40.8 ± 24.7	0.856
AST (U/L), mean ± SD	73.7 ± 68.4	51.6 ± 34.8	0.130
GGT (U/L), mean ± SD	234.9 ± 223.9	183.3 ± 211.8	0.368
INR, mean ± SD	1.2 ± 0.1	1.1 ± 0.1	0.092
AFP (ng/mL)			
< 400	13 (41.9%)	11 (39.3%)	0.836
≥ 400	18 (58.1%)	17 (60.7%)	
DCP (mAU/mL)			
< 400	3 (9.70%)	10 (35.7%)	0.036
≥ 400	28 (90.3%)	18 (64.3%)	
HBsAg			
–	05 (16.1%)	6 (21.4%)	0.602
+	26 (83.9%)	22 (78.6%)	
Antiviral therapies			
Entecavir	22 (84.6%)	18 (81.8%)	0.796
Tenofovir disoproxil fumarate	4 (15.4%)	4 (18.2%)	
HBV-DNA (IU/mL)			
≤ 1000	13 (41.9%)	21 (75.0%)	0.010
> 1000	18 (58.1%)	7 (25.0%)	

*AFP* alpha-fetoprotein ,*ALB* albumin, *ALT* alanine transaminase, *AST* aspartate transaminase, *BCLC* Barcelona Clinic Liver Cancer, *CNLC* China Liver Cancer, *DCP* des-γ-carboxy prothrombin, *GGT* gamma-glutamyl transpeptidase, *HBV* hepatitis B virus, *HbsAg* hepatitis B surface antigen, *INR* international normalized ratio, *NLR* neutrophil–lymphocyte ratio, *PS* performance status, *TB* total bilirubin, *WBC* white blood count

With respect to the forms of physical activity performed by patients in the active group, brisk walking was the most common one, accounting for 75.0%. In addition to brisk walk, 4 patients chose jogging (14.3%) and 3 patients chose ball games (3.6%), equipment exercising (3.6%) and swimming (3.6%) respectively (Additional file 2: Table S1).

### Regular physical activity was associated with improved OS and PFS

**Fig. 1:**
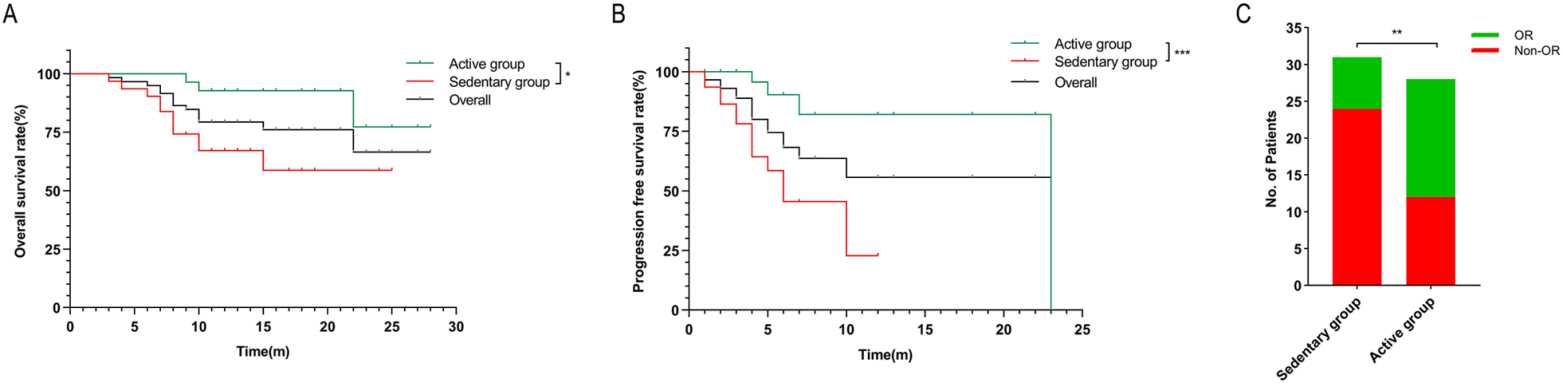
Regular physical activity was associated with improved outcomes in HCC patients receiving combined therapy. In a cohort of patients with unresectable or advanced HCC treated with lenvatinib plus anti-PD-1 antibody combination, regular physical activity was associated with an improved overall survival (**A**), progression-free survival (**B**), and high rate of objective response (**C**). * P < 0.05, ** P < 0.01, *** P < 0.001. *HCC* hepatocellular carcinoma, *OR* objective response

Of the 59 patients, 14 died and 17 experienced tumor progression by the last follow-up. The 1- and 2-year OS rates were 92.7% vs. 67.1% and 77.3% vs. 58.7% in the active group and sedentary group, respectively (P < 0.05, Fig. 1A). The 1-year PFS rate was 82.1% in the active group vs. 23.0% in the sedentary group (P < 0.001, Fig. 1B). The median PFS in the active group was 23 months, longer than that in the sedentary group (6 months, P < 0.001). The results of the univariate analysis are presented in Additional file 3: Table S2. Multivariate Cox regression analysis identified that physical activity was independently associated with improved OS (hazard ratio [HR] = 0.203, 95% confidence interval [CI], 0.052–0.794, P = 0.022) and PFS (HR = 0.158, 95% CI 0.044–0.562, P = 0.004) (Table 2).

**Table 2:** Multivariate analysis of the association between baseline factors and treatment outcomes

Variables	No. of patients	Overall survival		Progression-free survival		Objective response	
		Multivariate Cox regression				Multivariate Logistic regression	
		HR (95% CI)	P value	HR (95% CI)	P value	OR (95% CI)	P value
Regular physical activity							
Yes	28	0.203	0.022	0.158	0.004	4.204	0.016
No	31	(0.052–0.794)		(0.044–0.562)		(1.302–13.569)	
Macrovascular invasion							
Yes	34	5.430	0.03				
No	25	(1.183–24.935					
INR							
> 1.20	15					0.189	0.049
≤ 1.20	44					(0.036–0.992)	

*CI* confidence interval, *HR* hazard ratio, *INR* international normalized ratio, *OR* objective response

### Regular physical activity was associated with higher ORR

As of June 30, 2021, the ORR in the active group was 57.1% vs. 22.6% in the sedentary group (P < 0.01, Fig. 1C). The results of the univariate analysis are presented in Additional file 3: Table S1. Univariate and multivariate Logistic regression confirmed that regular physical activity and international normalized ratio (INR) were independently associated with OR in patients treated with combination therapy (odds ratio [OR] = 4.204, 95% CI 1.302–13.569, P = 0.016; OR = 0.189, 95% CI 0.036–0.992, P = 0.049, respectively) (Table 2).

### Physical activity improved the effect of combination therapy in syngeneic HCC mice

**Fig. 2:**
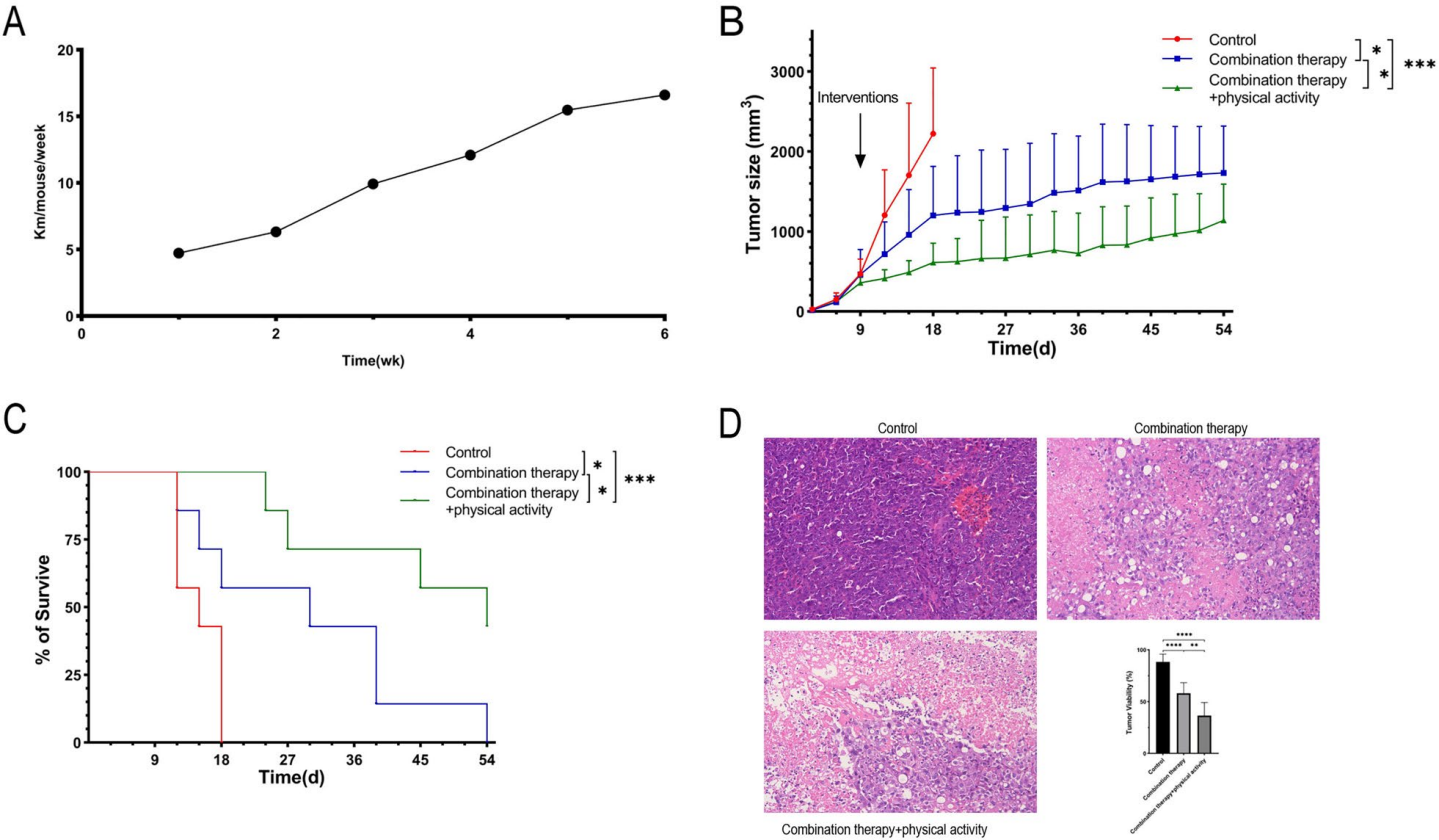
Voluntary running improved the effect of combination therapy in HCC mouse model. **A** Average running distance per mouse per week in the combination therapy plus physical activity group. **B** Tumor growth and **C** survival (time to 20 mm in maximum diameter) of subcutaneously implanted HCC mice. **D** Tumor viability assessed in Hematoxylin and eosin slides from the subcutaneous HCC mouse model. Representative images captured at 40 ×. * P < 0.05, **P < 0.01, ***P < 0.001, ****P < 0.0001. *HCC* hepatocellular carcinoma

To elucidate why physical activity improved the outcomes of HCC patients treated with combination therapy, we established a subcutaneous syngeneic HCC mouse model. The average running distance per mouse per week in the combination therapy plus physical activity group is shown in Fig. 2A. Compared to the control group, both combination therapy and combination therapy plus physical activity groups had lower tumor burden (P < 0.05 and P < 0.001, respectively; Fig. 2B). Physical activity synergized with combination therapy to further improve its inhibitory effect on tumor growth (P < 0.05; Fig. 2B).

Both combination therapy and combination therapy plus physical activity groups showed prolonged survival (time to 20 mm in maximum diameter) of tumor-bearing mice compared to the control group, and mice in the combination therapy plus physical activity group had the longest lifespan (median survival of 15, 30 and 54 days in control, combination therapy and combination therapy plus physical activity groups, respectively, P < 0.001; Fig. 2C).

Finally, tumor viability was assessed to further understand the synergistic effect of physical activity and combination therapy. The combination therapy plus physical activity group had the lowest tumor viability (P < 0.0001 vs. control group and P < 0.01 vs. combination therapy group), followed by the combination therapy group (P < 0.0001 vs. control group) (Fig. 2D).

### Mechanism of the synergistic effect of physical activity and combination therapy

**Fig. 3:**
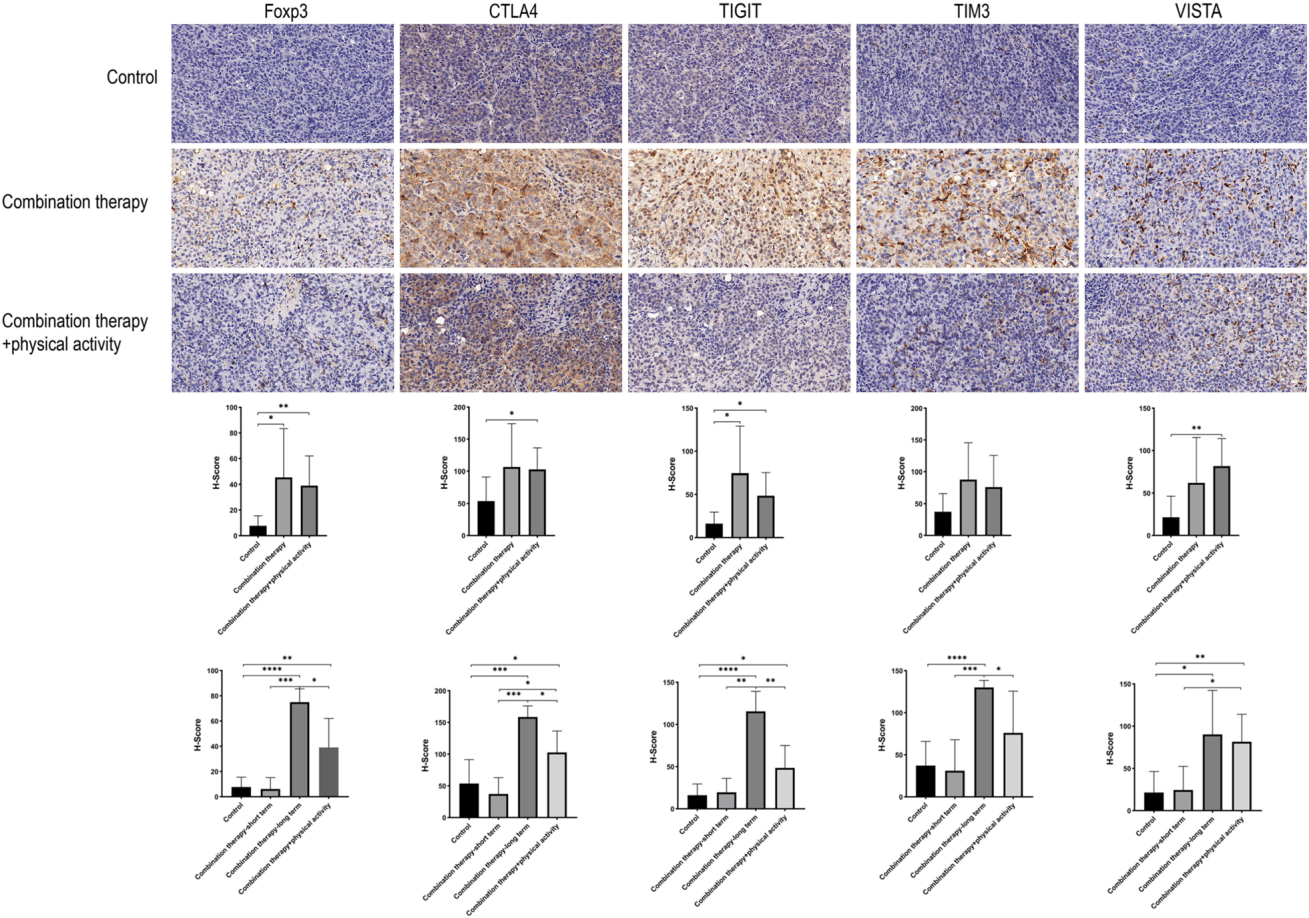
Immunohistochemical staining and analysis of subcutaneous tumors. Immunohistochemical staining was conducted on subcutaneous tumor tissue microarray. H-score were calculated to quantify the expression levels of marker proteins. Representative images captured at 40 ×. *P < 0.05, **P < 0.01, ***P < 0.001, ****P < 0.0001. *Foxp3* forkhead box protein p3, *CTLA4* cytotoxic T-lymphocyte-associated protein 4, *TIGIT* T cell immunoreceptor with Ig and ITIM domains, *TIM3* T-cell immunoglobulin mucin-3, *VISTA* V domain-containing Ig suppressor of T-cell activation

To elucidate why physical activity improved the efficacy of combination therapy, immunohistochemical staining of major immune cell populations and alternative immune checkpoints was performed. H-scores were generated to quantitatively analyze expression levels among the three groups. Both combination therapy and combination therapy plus physical activity groups showed increased CD4 + T and Treg cell infiltrations and TIGIT expression compared to the control group (Additional file 1: Fig. S1 & Fig. 3). The combination therapy plus physical activity group had elevated immune cell infiltration, including macrophage and CD8 + T cells, and increased CTLA4 and VISTA expression (SAdditional file 1: Fig. S1 & Fig. 3). Compared to the combination therapy group, the combination therapy plus physical activity group tended to exhibit reduced infiltration of Treg cells and expression of several immune checkpoints, including TIGIT and TIM3.

Interestingly, we observed that mice receiving short-term (< 15 days, n = 3) and long-term (≥ 15 days, n = 4) combination therapy showed distinct immunophenotypes. Short-term combination therapy had a similar immunophenotype to the control group, while long-term combination therapy exhibited an immunosuppressive phenotype, with increased infiltration of Treg cells and higher levels of alternative immune checkpoints, including CTLA4, TIGIT, TIM3 and VISTA (Fig. 3). However, the combination therapy plus physical activity group showed reduced infiltration of Treg cells and expression of CTLA4, TIGIT and TIM3 (Fig. 3).

Overall, we observed that physical activity inhibited the infiltration of immune-suppressive Treg cells and the adaptive upregulation of alternative immune checkpoints to enhance the therapeutic efficacy of combination therapy.

## Discussion

In this study, we found that regular physical activity was associated with improved outcomes in patients with unresectable HCC receiving combined lenvatinib and anti-PD-1 therapy. Compared with the sedentary population, physically active patients had an approximately 80% lower risk of death and progression and four times greater likelihood of reaching objective response criteria. Considering that patients’ basic general status might influence the intensity and frequency of physical activity, we excluded patients with performance score greater than 2. And multivariate analysis shown that the protective effect of regular physical activity was independent from patients’ basic general status. However, considering the small number of patients choosing physical activities other than brisk walk and the short follow-up period, it was difficult to explore the extent to which different physical activities affect survival. Which form of physical activity provides greater benefit to HCC patients required further research.

Previous treatment may affect the efficacy of physical activity and combination therapy. However, in the present study, lenvatinib plus anti-PD-1 was given as first-line systemic treatment, and only one patient in the active group received TACE before combination therapy. Thus, the effect of previous treatment could be excluded.

Ample studies have shown that physically active lifestyles reduce the risk of multiple cancers, including HCC [17, 18, 37]. Following primary treatment, physical activity has been consistently shown to have positive effects on vigor and vitality, cardiorespiratory fitness, quality of life, depression, anxiety, pain and fatigue; physical activity also reduces the overall risk of death and cancer-related death in cancer survivors [19–21, 38]. During primary treatment with lenvatinib, physical activity might enable patients to receive longer-term treatment through improvement of their physical and psychological condition [38]. Moreover, both physical activity and lenvatinib have immunomodulatory effects [15, 16, 25, 26]. Therefore, physical activity might synergize with lenvatinib to enhance the therapeutic efficacy of anti-PD-1 therapy.

To elucidate the reason why physical activity synergizes with combination therapy, we established a subcutaneously implanted HCC mouse model. Since mice are natural runners [39], running wheels were placed in cages of the combination therapy plus physical activity group to facilitate voluntary running. The mouse model showed that physical activity improved the therapeutic efficacy of combination therapy with retarded tumor growth and prolonged survival, consistent with clinical findings in humans.

We found that both combination therapy and combination therapy plus physical activity were associated with an immunosuppressive tumor microenvironment, which contradicts the findings of previous studies [15, 16]. However, the treatment duration in the present study was much longer (median treatment duration of 30 days) than those reported in previous studies [15, 16]. As short-term and long-term treatments showed distinct immunophenotypes, this may account for the contradictory findings of the present study. It is suggested that the immunostimulatory effect of lenvatinib might decrease with prolonged treatment duration.

Our findings suggest that physical activity enhances combined lenvatinib plus anti-PD-1 therapy by counteracting the immunosuppressive tumor microenvironment induced by long-term treatment. Compared to tumors treated with combination therapy, the addition of physical activity inhibited the infiltration of immunosuppressive Treg cells and the expression of several immune checkpoints, including CTLA4, TIGIT and TIM3, reprogramming the tumor immune microenvironment. Notably, the immunomodulatory function of physical activity during long-term treatment postponed tumor progression and prolonged OS. In addition to the modulation of immune microenvironment, physical activity ameliorates immunosenescence through promoting the secretion of several cytokines by skeletal muscle during physical activity, including IL-6, IL-7 and IL-15 [26]. Similarly, a meta-analysis conducted by Micael Deivison de Jesus Alves, et al. found that long-distance running was associated with increased levels of IL-6, IL-1ra, IL-1β, IL-8, IL-10, and TNF-α, and with decreased levels of IL-2, and IFN-γ [40]. In general, physical activity has immune-promoting effect, which may facilitate the anti-tumor immune response.

In addition to immunomodulatory effects, physical activity has been associated with other anti-cancer mechanisms. It was reported that physical activity suppressed tumor growth by promoting p53-driven apoptosis [41]. Our previous study found that moderate swimming inhibited liver cancer progression through suppression of transforming growth factor-beta-induced epithelial-mesenchymal transition [23]. It was also reported that exercise promoted a shift towards a more “normalized” tumor microenvironment by improving intra-tumoral perfusion/vascularization [42, 43]. Multiple mechanisms may therefore account for the anti-cancer effects of physical activity.

These findings add to the evidence supporting physical activity as an important lifestyle intervention in patients with cancer. In recent years, multiple international organizations have published physical activity recommendations for patients living with and beyond cancer, including the American Cancer Society [44], the American College of Sports Medicine [45], Cancer Care Ontario [46], and the Clinical Oncology Society of Australia [47], and Exercise and Sports Science Australia [48]. The specific value of physical activity in promoting an enhanced treatment response in patients receiving combination lenvatinib plus anti-PD-1 therapy should be explored further in this context.

Our study is not without limitations. Firstly, the retrospective design of the study may have introduced bias, such as recall bias and confounding bias. Secondly, the sample size of this retrospective study is small. Thus, a well-designed prospective study with large sample size is required to control bias and quantify the physical activity level. Thirdly, the PD-1 inhibitors used in this study was not unified. All PD-1 inhibitors were off-label therapies for HCC and cannot be reimbursed in China, which made patient’s choice become an important consideration (mostly cost and updated information from clinical trials). Fourthly, the duration of combination treatment might influence the intra-tumor immunophenotype, which was not accounted for at the time of study conception. Further studies are needed to confirm the effect of duration of combination treatment on the tumor microenvironment.

## Conclusions

The present study suggests that physical activity improved the therapeutic efficacy of long-term combined lenvatinib plus anti-PD-1 therapy in patients with HCC through reprograming the tumor microenvironment from an immunosuppressive to immunostimulatory phenotype. This study provides evidence for recommending physically active lifestyles to patients with unresectable HCC receiving combined lenvatinib and anti-PD-1 therapy.

## Supplementary Information

**Additional file 1: Figure S1.** Immunohistochemical staining and analysis of subcutaneous tumors. Immunohistochemical staining was conducted on subcutaneous tumor tissue microarray. H-score were calculated to quantify the expression levels of marker proteins. Representative images captured at 40X. *P < 0.05, **P < 0.01, ***P < 0.001, ****P < 0.0001.

**Additional file 2: Table S1.** Distribution of selected physical activities in the active group

**Additional file 3: Table S2.** Univariate analysis of the association between baseline factors and treatment outcomes. *AFP* alpha-fetoprotein, *ALB* albumin, *ALT* alanine transaminase, *AST* aspartate transaminase, *BCLC* Barcelona Clinic Liver Cancer, *CI* confidence interval, *CNLC* China Liver Cancer, *DCP* des-γ-carboxy prothrombin, *GGT* gamma-glutamyl transpeptidase, *HBV* hepatitis B virus, *HBsAg* hepatitis B surface antigen, *HR* hazard ratio, *INR* international normalized ratio, *NLR* neutrophil–lymphocyte ratio, *OR* odds ratio, *PS* performance status, *TB* total bilirubin *WBC* white blood count.

## Data Availability

The datasets used and/or analyzed during the current study are available from the corresponding author on reasonable request.

## Abbreviations

AFP: Alpha-fetoprotein
ALB: albumin
ALT: Alanine transaminase
AST: Aspartate transaminase
ATCC: American Type Culture Collection
BCLC: Barcelona Clinic Liver Cancer
CNLC: China Liver Cancer
CTLA4: Cytotoxic T-lymphocyte-associated protein 4
DCP: Des-γ-carboxy prothrombin
DMEM: Dulbecco’s modified eagle medium
ECOG: Eastern Cooperative Oncology Group
FBS: Fetal bovine serum
Foxp3: Forkhead box protein p3
GGT: Gamma-glutamyl transpeptidase
HBV: Hepatitis B virus
HbsAg: Hepatitis B surface antigen
HCC: Hepatocellular carcinoma
IACUC: Institutional Animal Care and Use Committees
ICB: Immune check point blockade
INR: International normalized ratio
ITIM: Immunoreceptor tyrosine-based inhibitory motif
mRECIST: Modified response evaluation criteria in solid tumors
NLR: Neutrophil–lymphocyte ratio
OR: Objective response
ORR: Objective response rate
OS: Overall survival
PD-1: Programmed cell death protein 1
PDL-1: Programmed cell death ligand 1
PFS: Progression free survival
PS: Performance status
TACE: Transarterial chemoembolization
TB: Total bilirubin
TIGIT: T cell immunoreceptor with Ig and ITIM domains
TIM3: T-cell immunoglobulin mucin-3
Treg cells: Regulatory T cells
VISTA: V domain-containing Ig suppressor of T-cell activation
WBC: White blood count
