# Supplementary figures and images for: Physical activity improves outcomes of combined lenvatinib plus anti-PD-1 therapy in unresectable hepatocellular carcinoma: a retrospective study and mouse model
Source: Exp Hematol Oncol. 2022 Apr 4;11:20. doi: 10.1186/s40164-022-00275-0 (PMC8978397; doi:10.1186/s40164-022-00275-0)

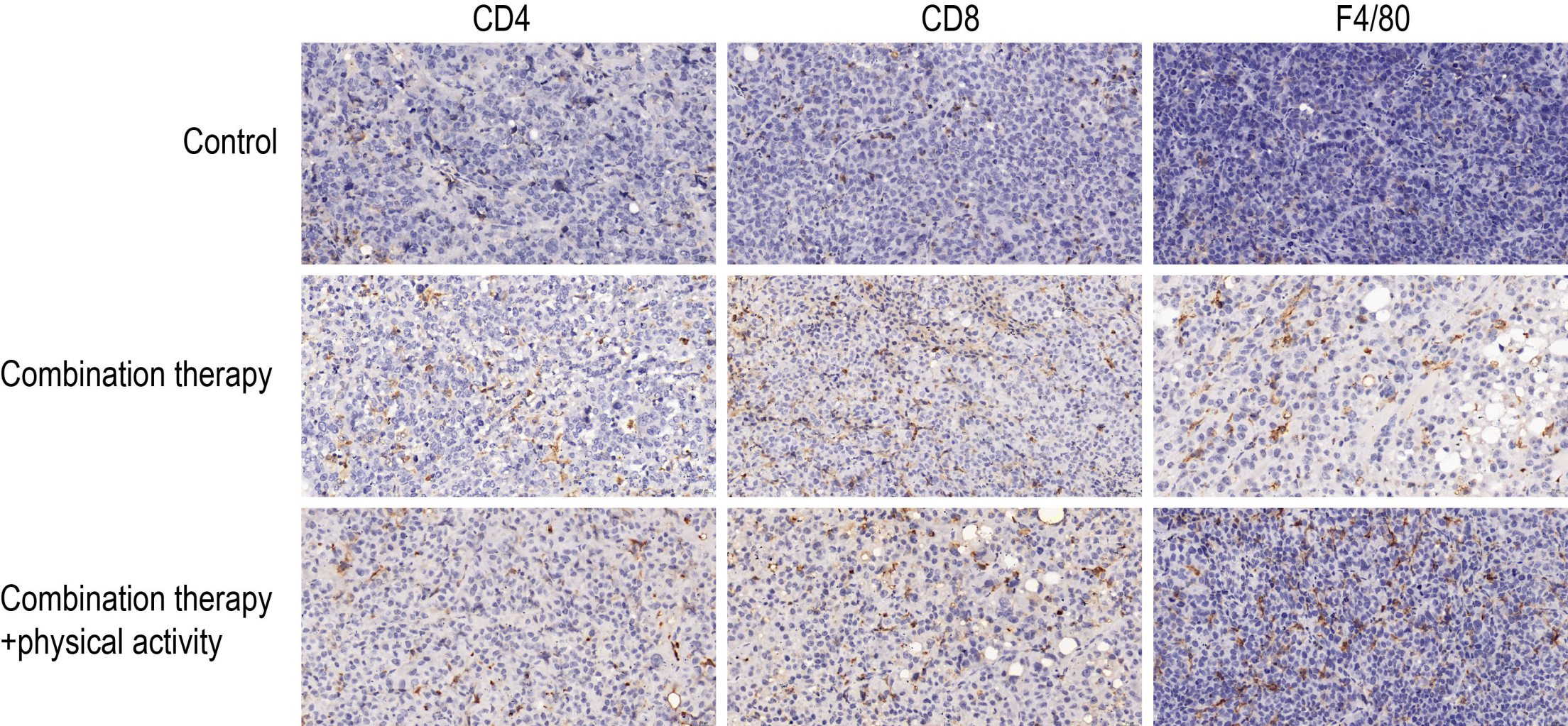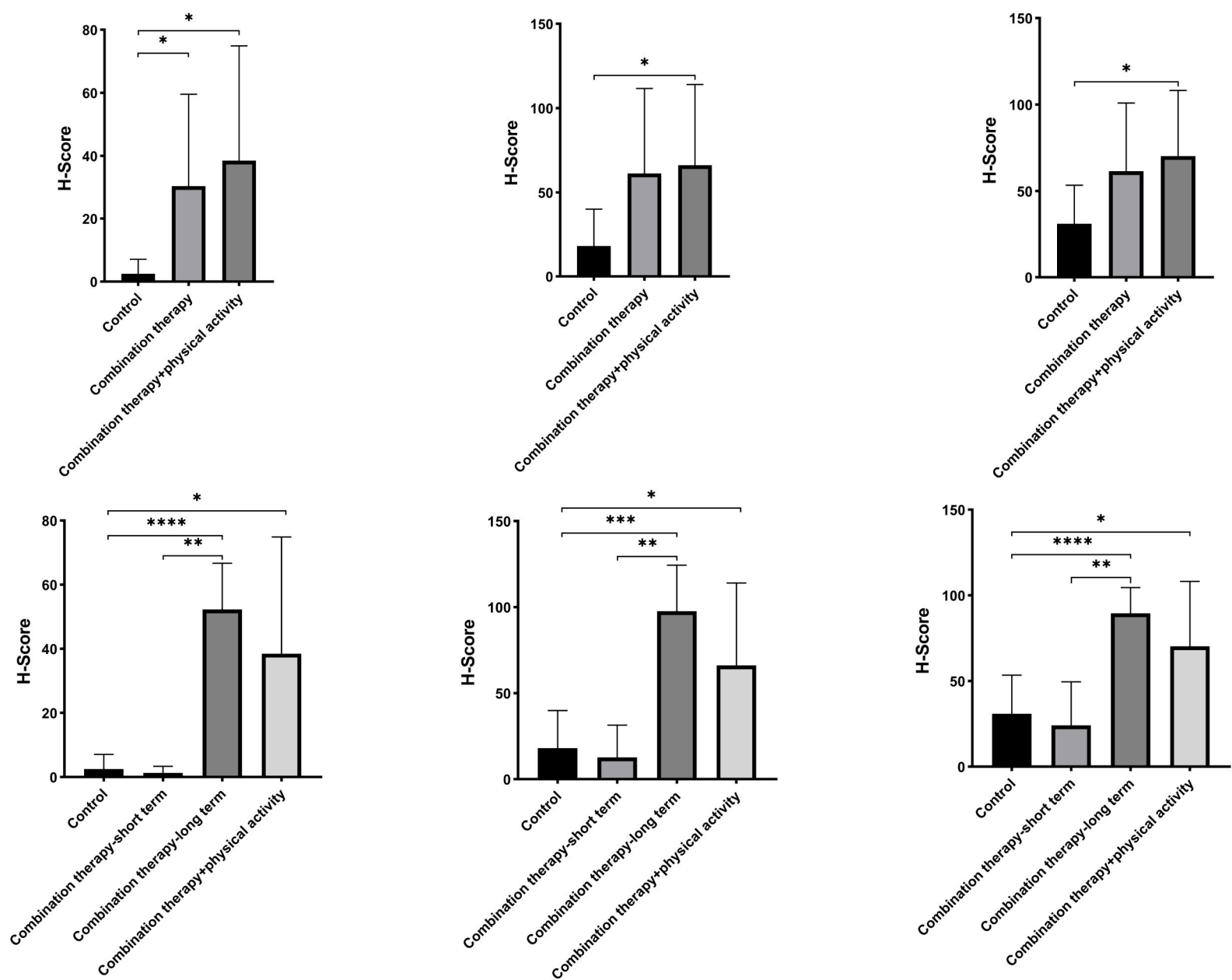

Supplement: Supplementary file 1 — Additional file 1: Figure S1. Immunohistochemical staining and analysis of subcutaneous tumors. Immunohistochemical staining was conducted on subcutaneous tumor tissue microarray. H-score were calculated to quantify the expression levels of marker proteins. Representative images captured at 40X. *P < 0.05, **P < 0.01, ***P < 0.001, ****P < 0.0001. [file 40164_2022_275_MOESM1_ESM.pdf]
