# Supplementary material for: Physical activity improves outcomes of combined lenvatinib plus anti-PD-1 therapy in unresectable hepatocellular carcinoma: a retrospective study and mouse model
Source: Exp Hematol Oncol. 2022 Apr 4;11:20. doi: 10.1186/s40164-022-00275-0 (PMC8978397; doi:10.1186/s40164-022-00275-0)
Supplement: Supplementary file 2 — Additional file 2: Table S1. Distribution of selected physical activities in the active group [file 40164_2022_275_MOESM2_ESM.docx]

**Supplementary table 1.** Distribution of selected physical activities in the active group

| **Physical activities** | **No. of patients** |
| --- | --- |
| Brisk walk | 21 (75.0%) |
| Jogging | 4 (14.3%) |
| Ball games | 1 (3.6%) |
| Equipment | 1 (3.6%) |
| Swimming | 1 (3.6%) |
| Sum | 28 (100%) |
