# Supplementary material for: Physical activity improves outcomes of combined lenvatinib plus anti-PD-1 therapy in unresectable hepatocellular carcinoma: a retrospective study and mouse model
Source: Exp Hematol Oncol. 2022 Apr 4;11:20. doi: 10.1186/s40164-022-00275-0 (PMC8978397; doi:10.1186/s40164-022-00275-0)
Supplement: Supplementary file 3 — Additional file 3: Table S2. Univariate analysis of the association between baseline factors and treatment outcomes. AFP alpha-fetoprotein, ALB albumin, ALT alanine transaminase, AST aspartate transaminase, BCLC Barcelona Clinic Liver Cancer, CI confidence interval, CNLC China Liver Cancer, DCP des-γ-carboxy prothrombin, GGT gamma-glutamyl transpeptidase, HBV hepatitis B virus, HBsAg hepatitis B surface antigen, HR hazard ratio, INR international normalized ratio, NLR neutrophil–lymphocyte ratio, OR odds ratio, PS performance status, TB total bilirubin WBC white blood count. [file 40164_2022_275_MOESM3_ESM.docx]

**Supplementary table 2.** Univariate analysis of the association between baseline factors and treatment outcomes

| **Variables** | **No. of patients** | **Overall survival** | | **Progression-free survival** | | **Objective response** | |
| --- | --- | --- | --- | --- | --- | --- | --- |
|  |  | **Univariate Cox regression** | | | | **Univariate Logistic regression** | |
|  |  | **HR (95% CI)** | **P value** | **HR (95% CI)** | **P value** | **OR (95% CI)** | **P value** |
| Age (years)  >50  ≤50 | 38  21 | 2.442  (0.673 - 8.865) | 0.157 | 1.302  (0.452 - 3.753) | 0.618 | 1.060  (0.355 - 3.168) | 0.917 |
| Sex  Male  Female | 53  6 | 1.957  (0.432 - 8.857) | 0.369 | 3.165  (0.876 - 11.433) | 0.059 | 0.762  (0.128 - 4.538) | 0.765 |
| Regular physical activity  No  Yes | 31  28 | 0.220  (0.060 - 0.799) | 0.011 | 0.158  (0.044 - 0.562) | 0.001 | 4.571  (1.482 - 14.102) | 0.008 |
| Overweight  No  Yes | 56  3 | 1.083  (0.140-8.363) | 0.939 | 0.045  (0.000-1116.628) | 0.549 | 0.000 | 0.999 |
| PS score  0  1 | 30  29 | 4.438  (1.235 - 15.947) | 0.012 | 1.314  (0.489 - 3.531) | 0.580 | 0.278  (0.092 - 0.847) | 0.024 |
| BCLC stage  A  B  C | 2  15  42 | 27.739  (0.452 - 1703.199) | 0.114 | 1.272  (0.483 - 3.350) | 0.725 | 0.864  (0.328 - 2.277) | 0.767 |
| CNLC stage  I  II  III | 4  13  42 | 21.158  (0.485 - 922.706) | 0.113 | 1.346  (0.543 - 3.338) | 0.538 | 0.855  (0.363 - 2.013) | 0.720 |
| Extra-hepatic metastasis  No  Yes | 47  12 | 1.103  (0.306 - 3.979) | 0.880 | 1.034  (0.291 - 3.673) | 0.959 | 1.765  (0.491 - 6.337) | 0.384 |
| Macrovascular invasion,  No  Yes | 25  34 | 5.203  (1.163 - 23.266) | 0.015 | 1.177  (0.436 - 3.179) | 0.744 | 0.518  (0.179 - 1.501) | 0.226 |
| Child-Pugh classification,  A  B | 57  2 | 0.047  (0.000 - 25547.589) | 0.487 | 0.048  (0.000 - 59236.183) | 0.513 | 1.591  (0.095 - 26.761) | 0.747 |
| Tumor size (cm)  <11.5  >11.5 | 30  29 | 2.127  (0.709 - 6.375) | 0.164 | 1.326  (0.494 - 3.558) | 0.568 | 0.688  (0.240 - 1.972) | 0.487 |
| WBC (*10^9/L)  <3.5  3.5 - 9.5  >9.5 | 7  49  3 | 1.155  (0.561 - 2.378) | 0.695 | 0.948  (0.433 - 2.076) | 0.894 | 2.008  (0.889 - 4.535) | 0.094 |
| NLR |  | 1.393  (1.006 - 1.928) | 0.046 | 0.952  (0.599 - 1.515) | 0.836 | 0.818  (0.556 - 1.204) | 0.309 |
| TB (μmol/L)  ≤20.4  >20.4 | 40  19 | 1.386  (0.478 - 4.018) | 0.542 | 0.531  (0.151 - 1.865) | 0.308 | 1.212  (0.398 - 3.690) | 0.735 |
| ALB (g/dL)  ≥35  <35 | 32  27 | 1.309  (0.457 - 3.751) | 0.611 | 1.795  (0.667 - 4.830) | 0.233 | 0.860  (0.300 - 2.463) | 0.778 |
| AST ALT ratio,  >1  <1  ≈1 | 26  10  23 | 1.796  (0.935 - 3.452) | 0.162 | 1.115  (0.649 - 1.915) | 0.911 | 0.696  (0.389 - 1.248) | 0.224 |
| GGT (U/L)  ≤45  >45 | 10  49 | 27.186  (0.070 - 10593.674) | 0.278 | 0.946  (0.268 - 3.340) | 0.930 | 0.950  (0.237 - 3.812) | 0.942 |
| INR  ≤1.20  >1.20 | 44  15 | 1.318  (0.404 - 4.305) | 0.642 | 2.869  (1.010 - 8.151) | 0.035 | 0.168  (0.034 - 0.836) | 0.029 |
| HBsAg  ( - )  (+) | 11  48 | 1.391  (0.311 - 6.234) | 0.661 | 1.495  (0.339 - 6.588) | 0.587 | 1.147  (0.295 - 4.458) | 0.844 |
| AFP (ng/mL)  <400  ≥400 | 24  35 | 1.192  (0.399 - 3.560) | 0.751 | 0.742  (0.278 - 1.981) | 0.544 | 1.111  (0.382 - 3.232) | 0.847 |
| DCP (mAU/mL)  <400  ≥400 | 13  46 | 1.138  (0.317 - 4.081) | 0.841 | 0.723  (0.229 - 2.288) | 0.573 | 1.029  (0.290 - 3.643) | 0.965 |
| HBV-DNA (IU/mL)  ≤1000  >1000 | 34  25 | 2.596  (0.868 - 7.765) | 0.074 | 1.556  (0.582 - 4.162) | 0.367 | 0.596  (0.203 - 1.754) | 0.347 |

**Abbreviations:** AFP, alpha-fetoprotein; ALB, albumin; ALT, alanine transaminase; AST, aspartate transaminase; BCLC, Barcelona Clinic Liver Cancer; CI, confidence interval; CNLC, China Liver Cancer; DCP, des-γ-carboxy prothrombin; GGT, gamma-glutamyl transpeptidase; HBV, hepatitis B virus; HbsAg, hepatitis B surface antigen; HR, hazard ratio; INR, international normalized ratio; NLR, neutrophil-lymphocyte ratio; OR, odds ratio; PS, performance status; TB, total bilirubin, WBC, white blood count.
